# Use and Appreciation of a Tailored Self-Management eHealth Intervention for Early Cancer Survivors: Process Evaluation of a Randomized Controlled Trial

**DOI:** 10.2196/jmir.5975

**Published:** 2016-08-23

**Authors:** Iris Maria Kanera, Roy A Willems, Catherine A W Bolman, Ilse Mesters, Victor Zambon, Brigitte CM Gijsen, Lilian Lechner

**Affiliations:** ^1^ Faculty of Psychology and Educational Sciences Open University of the Netherlands Heerlen Netherlands; ^2^ Research Institute CAPHRI Department of Epidemiology, Optimizing Patient Care Maastricht University Maastricht Netherlands; ^3^ Department of Urology Zuyderland Medical Center Heerlen and Sittard-Geleen Netherlands; ^4^ Netherlands Comprehensive Cancer Organisation (IKNL) Utrecht Netherlands

**Keywords:** eHealth, web-based intervention, computer tailoring, cancer survivorship, intervention usage, appreciation, multiple behavior intervention, process evaluation, self-management

## Abstract

**Background:**

A fully automated computer-tailored Web-based self-management intervention, Kanker Nazorg Wijzer (KNW [Cancer Aftercare Guide]), was developed to support early cancer survivors to adequately cope with psychosocial complaints and to promote a healthy lifestyle. The KNW self-management training modules target the following topics: return to work, fatigue, anxiety and depression, relationships, physical activity, diet, and smoking cessation. Participants were guided to relevant modules by personalized module referral advice that was based on participants’ current complaints and identified needs.

**Objective:**

The aim of this study was to evaluate the adherence to the module referral advice, examine the KNW module use and its predictors, and describe the appreciation of the KNW and its predictors. Additionally, we explored predictors of personal relevance.

**Methods:**

From the respondents (N=231; mean age 55.6, SD 11.5; 79.2% female [183/231]), 98.3% (227/231) were referred to one or more KNW modules (mean 2.9, SD 1.5), and 85.7% (198/231) of participants visited at least one module (mean 2.1, SD 1.6). Significant positive associations were found between the referral to specific modules (range 1-7) and the use of corresponding modules. The likelihoods of visiting modules were higher when respondents were referred to those modules by the module referral advice. Predictors of visiting a higher number of modules were a higher number of referrals by the module referral advice (β=.136, *P*=.009), and having a partner was significantly related with a lower number of modules used (β=-.256, *P*=.044). Overall appreciation was high (mean 7.5, SD 1.2; scale 1-10) and was significantly predicted by a higher perceived personal relevance (β=.623, *P*=.000). None of the demographic and cancer-related characteristics significantly predicted the perceived personal relevance.

**Results:**

The KNW in general and more specifically the KNW modules were well used and highly appreciated by early cancer survivors. Results indicated that the module referral advice might be a meaningful intervention component to guide the users in following a preferred selection of modules. These results indicate that the fully automated Web-based KNW provides personal relevant and valuable information and support for early cancer survivors. Therefore, this intervention can complement usual cancer aftercare and may serve as a first step in a stepped-care approach.

**Conclusions:**

The KNW in general and more specifically the KNW modules were well used and highly appreciated by early cancer survivors. Indications were found that the module referral advice might be a meaningful intervention component to guide the users in following a preferred selection of modules. These results indicate that the fully automated Web-based KNW provides personal relevant and valuable information and support for early cancer survivors. Therefore, this intervention can complement usual cancer aftercare and may serve as a first step in a stepped-care approach.

**Trial Registration:**

Nederlands Trial Register: NTR3375; http://www.trialregister.nl/trialreg/admin/rctview.asp?TC=3375 (Archived by WebCite at http://www.webcitation.org/6jo4jO7kb)

## Introduction

Recovery from cancer and its treatment can be challenging for cancer survivors. A variety of physical, psychosocial, and lifestyle difficulties might impede the resumption of previous daily life activities [1]. Cancer aftercare guidelines for oncology professionals recommend paying attention to the early detection and recognition of psychological distress, fatigue, pain, problems with daily activities, lifestyle risks, and also to stimulating self-care within the first year after completing the primary curative cancer treatment [2,3]. Further, due to the aging population and improved cancer care, the population of cancer survivors is growing while complaints, needs, and preferences of cancer survivors can vary individually over the different subjects and time [4-7]. For these reasons, fully automated, computer-tailored Web-based cancer aftercare interventions may be suitable for providing a large number of cancer survivors with personalized advice at relatively low costs [8]. Moreover, online solutions fit well with the increasing numbers of cancer survivors who search the Internet for health-related information, especially with those survivors who do not seek face-to-face guidance or treatment [9,10]. Web-based interventions might be appropriate to be integrated as a first step in a stepped-care approach as it offers a low-intensive intervention first before referring to interventions that are more intensive. Such first-step, low-intensive interventions might be sufficient to meet the personal needs of a large proportion of survivors with relatively mild complaints and are less costly [11]. In addition, Web-based interventions can comprise relevant information as written text, videos, animations, interactive features, hyperlinks, while personalization of the content is possible by applying computer tailoring [12-14].

**Figure 1:**
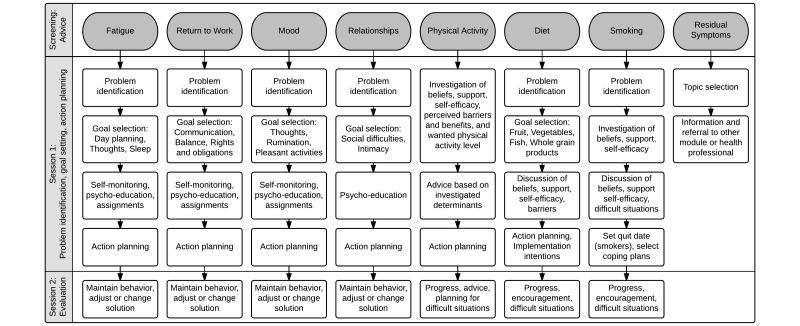
Overview of the scope and sequence of the modules. From Willems et al (2015). Used with permission.

**Figure 2:**
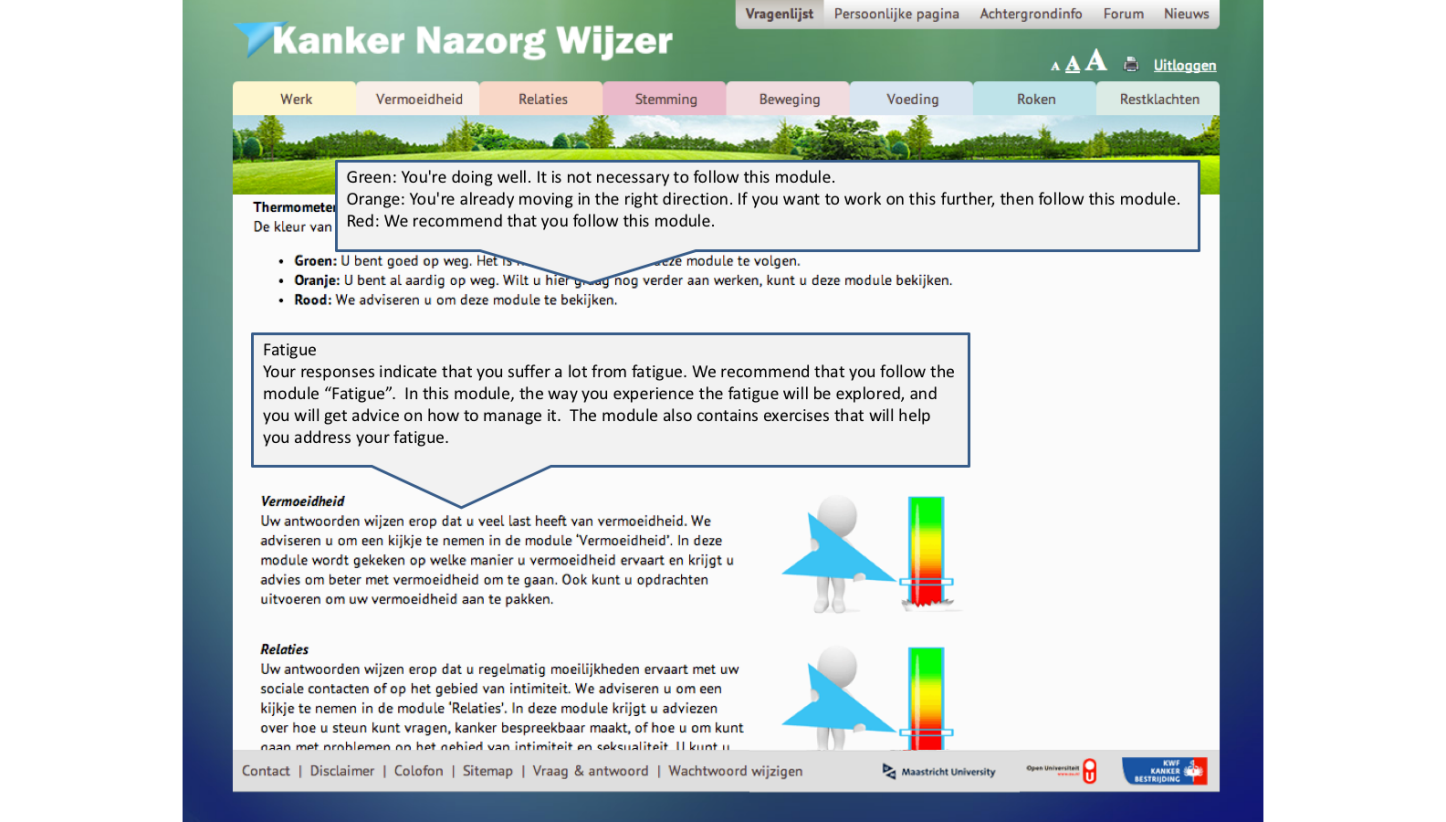
Module Referral Advice that encourages participants to follow relevant KNW modules. Adapted from Willems et al (2015). Used with permission.

The Web-based intervention *Kanker Nazorg Wijzer* (Cancer Aftercare Guide, KNW) is a fully automated intervention that aims to increase survivors’ quality of life (QoL) by providing psychosocial support as well as promoting positive lifestyle changes, and it targets cancer survivors of any type of cancer [15]. The KNW consists of seven self-management training modules covering the topics return to work, fatigue, anxiety and depression, social relationship and intimacy issues, physical activity, diet, and smoking cessation (see Figure 1), supplemented with one general information module on residual symptoms. Based on the responses to a screening questionnaire, cancer survivors receive personalized advice on which KNW modules are most relevant for them to use. This Module Referral Advice (MRA) is designed in a fashion analogous to traffic lights as displayed in Figure 2. This MRA aims to guide participants through the wide-ranging KNW portal, based on experienced complaints and identified needs, as assessed by the screening questionnaire. The KNW has been shown to be effective in reducing fatigue and depressive symptoms and in improving quality of life domains (ie, emotional and social functioning) [16]. In addition, strong indications were found that KNW users are engaged in more moderate physical activity and have a higher intake of vegetables, fruits, and fish 6 months after they started using the KNW [17]. Besides assessing the effects of the KNW, it is important to understand how this complex intervention was used and appreciated by the participants, whether use and appreciation was predicted by certain user characteristics, and to evaluate relevant key intervention components [8,18-20]. Moreover, it is essential to examine specifically whether the provided information was perceived as personally relevant in order to evaluate the computer tailoring.

Previously published Web-based interventions in the areas of lifestyle, mental health, and chronic conditions differ with regard to the number of (cancer-related) topics, the composition of the target group, the intervention components, and the delivery mode [8,21-25]. Generally, typical Web-based interventions are modular in set-up, are updated weekly, require weekly visits, last for about 10 weeks, and include interaction with the system, peers, or a counselor [26]. The actual use of most interventions was low, or data on the use have been poorly reported [8,26]. The extent of use might be influenced by differences in participant and intervention characteristics [27]. Prior studies among cancer survivors have shown that different user characteristics were related to different user patterns: for example, a higher usage was found among those with low levels of self-reported social support and a high illness burden, and among survivors who were working and who received radiotherapy [28,29]. Being female, middle aged or older, having mid to high levels of education, a healthy body mass index (BMI), a healthier lifestyle, and having a low quality of life were predictors for a higher use of (multiple behavior) eHealth interventions among the general population [30,31]. Reported intervention characteristics that might predict usage were peer or counselor support, in-person contact, updates of the intervention, and sending reminders [20,26,27]. According to previously published studies, mixed results were found on the relationship between intervention usage and outcomes, such as symptom distress, depression, and lifestyle behaviors [29,32,33]. With regard to appreciation, prior studies reported that Web-based interventions were positively evaluated by cancer survivors, and a higher use was associated with a higher appreciation in a generic Web-based intervention for breast cancer survivors [24,34,35].

The design of the KNW portal differs from most of the existing Web-based interventions for cancer survivors by providing personalized self-management training on seven topics and by allowing users to choose which modules they want to use during an intervention period of 6 months. Previously identified effective intervention characteristics of Web-based lifestyle interventions were tailored feedback, the use of theory, interactivity, goal setting, and online or in-person contact [8,26]. The KNW comprises all these elements, except for in-person contact. However, the MRA provides automated personalized guidance through the KNW modules. Given the large scope and the varied target group of the KNW portal, it is important to assess how the intervention was used, appreciated, whether the content was sufficiently tailored to be perceived as personal relevant, and what possible factors, including personal relevance, might predict the module use and its appreciation. In addition, the MRA might be a meaningful intervention component; therefore, the association between the MRA and the KNW module use also needs to be evaluated.

The main objective of this study is threefold: (1) to describe the use of the KNW modules and to identify predictors of a higher number of modules used, (2) to investigate the adherence to the provided MRA, and (3) to describe the appreciation of the KNW and its predictors. Additionally, to explore how well the tailoring worked and whether the perceived personal relevance might be different among subgroups, we explored possible predictors of personal relevance.

## Methods

This process evaluation was conducted as part of a two-armed randomized controlled trial (RCT) that evaluates the effects of the KNW portal. For the purpose of this report, all respondents of the intervention condition were included in the analyses. The details of the trial design, sample size calculation, participant eligibility, recruitment procedures, and the intervention have been published elsewhere [15-17]. Ethical approval for this trial (Dutch Trial Register NTR3375) was obtained from the Medical Research Ethics Committee, METC Z (NL41445.096.12, 12-T-115). All procedures performed in this study were in accordance with the ethical standards of the institutional and national research committee and with the 1964 Helsinki declaration and its later amendments of comparable ethical standards. Informed consent was obtained from all individual participants included in the study.

### Specific Intervention Elements: Module Referral Advice and Module Principles

A comprehensive description of the intervention, including the eight KNW modules, the underlying theoretical frameworks, and technical features are published in detail elsewhere [15,17]. This section describes the details of the MRA that was based on personal scores from the baseline questionnaire and that can refer to the seven self-management modules of the KNW (see Figure 2). The classification criteria for green, orange, and red MRA are summarized in Table 1 [36-45]. A green MRA signifies that the respondent reported no complaints, or minor complaints or needs, concerning the specific topic. Therefore, following the correspondent module is not a high priority. An orange MRA was provided when the respondents reported elevated but not severe complaints, or when respondents partially adhered to the lifestyle recommendations of the World Cancer Research Fund/American Institute for Cancer Research and the American Cancer Society [46,47]. The orange advice praises respondents’ reasonably positive scores; however, it is recommended that they follow the corresponding module for further improvement. This orange category includes a wide coverage of score ranges, allowing for participants with higher, but not severe scores to still receive some positive and encouraging feedback and not lose their motivation to follow a module due to feedback that might be perceived as too stringent. A red MRA was provided only when severe psychosocial complaints, problematic functioning, or low/no adherence to lifestyle recommendations was reported, thus indicating that the respondent might be in high need of support concerning the specific topic. In that case, it was strongly recommended to follow the corresponding module. More detailed information on the underlying measures and cut-off points is included in Multimedia Appendix 1.

**Table 1:** Classification of the green, orange, and red MRA.

	Measurements and classification criteria^a^	MRA categories		
		Green	Orange	Red
Fatigue	CIS, subscale subjective fatigue (1-56) [36]	<27	27-35	>35
Return to work	Extended CaSUN [37,38]: Needs to adjust/ find a job (0-5); Needs to receive financial support (0-5); Needs support up on returning to work (0-5); Needs legal information (0-5)	No needs	Score on needs 3-12	Score on needs ≥13
Mood	HADS-A (0-21); HADS-D (0-21) [39]; MAC: dimension negative adjustment to cancer (16-64) [40]	HADS-A<8 and HADS-D<8 and MAC ≤36	HADS-A < 8 and HADS-D 8-15 and/ or MAC > 36; HADS-A 8-15 and HADS-D <8 or 8-15	HADS-A < 8 or 8-15 and HADS-D >15; HADS-D < 8 or 8-15 and HADS-A >15
Relationships	SSL-D (6-24) [41]/ CaSUN (2 items^b^)	SSL-D ≤7	SSL-D=8 or 9 & needs CaSUN	SSL-D ≥10 & needs CaSUN
Physical activity	SQUASH [42,43]: Weekly **≥**150 min moderate to vigorous PA; Daily ≥30 min of moderate PA on **≥**5 days p/w	Meeting both conditions	Meeting 1 out of 2 conditions	Meeting no conditions	
Diet	Dutch Standard Questionnaire on Food Consumption [44]: Daily ≥200g vegetables; Daily ≥2 pieces of fruit; Weekly ≥2 servings of fish; Daily ≥15g whole grains^c^; Daily ≥4 servings of potatoes/ whole-grain rice/ whole-grain pasta	Meeting at least 4 out of 5 conditions	Meeting 2 or 3 out of 5 conditions	Meeting 1 or 0 out of five conditions	
Smoking	Smoking, not smoking, time point of quitting [45]	Never/formersmokers, quit prior to cancer diagnosis	Quit smoking after cancer diagnosis	Current smokers

^a^CIS: Checklist Individual Strength; PA: physical activity; HADS: Hospital Anxiety and Depression Scale, HADS-A: subscale anxiety, HADS-D: subscale depression; MAC: Mental Adjustment to Cancer Scale; SSL-D: Social Support List‒discrepancy subscale; SQUASH: Short Questionnaire to Assess Health Enhancing Physical Activity

^b^Needs related to sexuality and fertility.

^c^Whole-grain bread, oatmeal, cereals.

Throughout the different KNW intervention modules, principles of problem-solving therapy, cognitive behavioral therapy, social cognitive theories, and self-regulation theories were applied [48-51]. According to the I-Change Model [50], awareness factors such as knowledge, cues to action, and risk perception might be important determinants in the dynamic process of behavior change by influencing motivation and intention. By applying the MRA, participants were made aware of their current psychosocial status and lifestyle behaviors in relation to the norms and guidelines, with the aim of guiding the participants toward the appropriate self-management modules. When using the modules, self-management skills training was provided by encouraging respondents to observe their current behavior more in detail, choose themes to work on, set goals, and to prepare action and coping plans, followed by monitoring their experiences and possible progress in the changed strategies and behaviors. Within the modules, the information and support was tailored to the current emotional status, lifestyle behavior, and motivational determinants (attitude, self-efficacy, intention) by the application of computer tailoring. Furthermore, the feedback was tailored to personal characteristics (gender, age, marital status, children, education level), and cancer-related and medical issues (type of cancer, comorbidities). Four weeks after completing (parts of) one module, the participants were invited to reflect on their behavioral change plans and experiences in a brief personalized evaluation session. They were also encouraged to continue applying the previously recommended self-management skills. Furthermore, valuable generic information about lifestyle and psychosocial issues was accessible when visiting the user forum and the monthly news items.

### Measurements

All data were derived from online self-report questionnaires and logging details.

**Module Use**

Module use was assessed by using logging data. Actual use was dichotomized (yes/no) for each module separately (in total eight modules). Module use was categorized into “yes” when at least the first three pages of a module were used. These three pages comprised important key information after which participants followed personalized pathways through the modules. The individual pathways were based on the responses to the baseline questionnaire, own preferences and goals, and take into consideration that the amount of needed information and/or support can vary to initiate behavior change [33]. Additionally, by assessing login data (last day the separate modules were used), the number of weeks of module engagement was registered.

#### Appreciation

At 6-month follow-up, the overall rating of the KNW and separate ratings for each of the used module(s) were assessed on a scale ranging from 1 (very poor) to 10 (outstanding) (eg, “Overall, how do you rate the KNW? Select your rating (1-10)”; “How do you rate module mood on a scale from 1 to 10”). Further, four separate items were measured to evaluate whether the provided information and support was understandable, useful, personally relevant, and recommendable to fellow patients, on a 5-point Likert-scale, ranging from 1 (low) to 5 (high). The perceived personal relevance (“Was the information from the Kanker Nazorg Wijzer of personal relevance for you?”) was included in the analysis of this study to explore whether computer tailoring worked well within the KNW. These items correspond to items that were used in other studies to measure the appreciation of Web-based interventions [52-54].

#### Demographic and Cancer-Related Variables

Information about demographic and cancer-related characteristics was collected at baseline. Standard questions were used to measure age, gender, and marital status. Marital status was dichotomized into “with partner” (married, cohabiting partners) and “without partner” (single, divorced, widowed). Education level was categorized into “low” (lower vocational education, medium general secondary education), “medium” (secondary vocational education, higher general secondary education), and “high” (higher vocational education, university education). Employment status was dichotomized into “working” (self-employed, in paid employment) and “not working” (unemployed, retired, unable to work). Type of cancer was categorized into breast, colorectal, and other types of cancer (ie, bladder, esophageal, gynecologic, hematologic, kidney, liver, lung, prostate, stomach, testicular, and thyroid cancer). Type of treatment was categorized into surgery and chemotherapy and radiotherapy, surgery and chemotherapy, surgery and radiotherapy, and other types of treatment. Further, aftercare (yes/no) and comorbidities (yes/no) were measured, and height and weight were assessed to determine BMI. The time since completion of primary treatment in weeks was based on registry data from the hospitals.

### Statistical Analyses

The analyses were performed using STATA version 13.1. Descriptive statistics were used to describe demographic and cancer-related characteristics of the module (non-) users and the number of weeks of module engagement among all participants of the intervention condition at baseline. To calculate the appreciation outcomes, participants who completed the relevant questions at the 6-month measurement and who used the corresponding modules were included. Chi-square tests were used to determine the relationships between the MRA and the subsequent module use with a two-sided alpha=.05 level of significance. Negative binominal regression analysis was used to identify the predictors of a higher number of modules used (0-8), due to overdispersed count data. Independent variables (hypothesized predictors) were demographic variables (gender, age, marital status, education, employment), cancer-related variables (cancer type, type of treatment, number of weeks after completing primary cancer treatment, aftercare, comorbidities, BMI), the number of red and orange MRA, ranging from 0-7, and the perceived personal relevance, ranging from 1-5. To examine the predictors of a higher overall appreciation of the KNW, multiple linear regression analysis was applied among participants who completed the follow-up questionnaire after 6 months. The dependent variable was the overall rating of the KNW, measured at 6-month follow-up, ranging from 1-10. The same independent variables as described above were counted as predictors. Furthermore, the number of used modules (sum score 0-8) was added to the multiple linear regression model. To explore possible predictors of perceived personal relevance, ordered logistic regression analysis was conducted, taking into consideration that the dependent variable, perceived personal relevance, was an ordinal variable, ranging from 1-5. Within this analysis, all demographic and cancer-related characteristics were added as independent variables. Dummy coding was used for categorical variables including more than two categories and the continuous and ordinal variables were standardized in all conducted regression analyses. Since filling out all computer-based questions was required, and respondents were reminded automatically if a question was not answered, there were no missing data at baseline. Missing data at 6-month follow-up due to dropout were not imputed when calculating appreciation outcomes.

## Results

Baseline characteristics of the intervention participants are displayed in Table 2. The majority of the participants was female (79.2%, 183/231), mean age was 55.6 (SD 11.5) years, and 70.1% (162/231) had been treated for breast cancer. A detailed overview of cancer diagnoses among the sample is shown in Multimedia Appendix 2. Mean time since completing primary cancer treatment was 25.1 (SD 13.5) weeks.

### Module Use

The majority (80-100%) of the module users continued after reading the first three compulsory pages of the different modules. The numbers and percentages of participants who used the separate modules are displayed in Table 2. The diet module (134/231, 58.0%) was used most often, and the smoking module was used least often (23/231, 10.0%). However, from all the smokers at baseline (n=27), 13 (48%) individuals used the module Smoking. Overall, the participants used on average 2.1 (SD 1.6) KNW modules; 14.3% (33/231) used no modules, 30.3% (70/231) used one module, 18.2% (42/231) used two modules, 21.2% (49/231) used three modules, 8.7% (20/231) used four modules, 3.9% (9/231) used five modules, and 3.4% (8/231) individuals used six or more modules. Module engagement was highest during the first 16 weeks after getting KNW access: around 80% of the users used the modules within this period.

### Provided Module Referral Advice

Table 3 displays how the red, orange, and green MRA ranged among the participants and how the modules were used. For fatigue, diet, and smoking, more red compared to orange MRA was provided, and for return to work, mood, relationships, and PA, more orange compared to red MRA was given. Green MRA was most frequently given with regard to smoking, return to work, mood, and relationships. Module use after getting a red or orange MRA was 58.8% and 38.6% for module fatigue, 55.6% and 52.4% for module return to work, 25% and 30.3% for module mood, and 25.9% and 27.3% for module relationships. Concerning the lifestyle modules, module use after receiving a red or orange MRA for PA was 25% and 35%, for diet 50.4% and 68.7%, and for smoking 48.2% and 42.9%. From the 231 participants, 173 (74.9%) received at least one red MRA, and 192 (83.1%) received at least one orange MRA. On average, the participants were referred to 2.9 (SD 1.5) relevant modules (either red or orange MRA, not displayed).

### Adherence to the Provided Module Referral Advice

The relations between the color of MRA (respectively red, orange, green) and module use are shown in Table 4. In general, the likelihood that participants actually used a relevant module was higher when the MRA was red or orange compared to green. When comparing module use after receiving a red MRA versus an orange MRA for the modules return to work, mood, relationships, PA, smoking, the differences were small, meaning that both colors led to comparable module participation. Participants used modules Fatigue (*X*^2^=4.599, *P=*.032, OR 2.262) more often when a red MRA was provided compared to an orange MRA. The diet module (*X*^2^=7.553, *P=*.006, OR .463) was used more often when an orange MRA was provided compared to a red MRA.

**Table 2:** Overall baseline characteristics of the KNW participants and categorized for module use (N=231).

		Overall (N=231)	No module (n=33, 14.3%)	KNW Modules							
				Fatigue (n=82, 35.5%)	Return to work (n=53, 22.9%)	Mood (n=49, 21.2%)	Relation-ships (n=38, 16.5%)	Physical activity (n=51, 22.1%)	Diet (n=134, 58%)	Smoking (n=23, 10%)	Residual symptoms (n=47, 20.4%)
Female, n (%)		183 (79.2)	26 (78.8)	63 (76.8)	46 (86.8)	41 (83.7)	30 (79.0)	44 (86.3)	106 (79.1)	17 (73.9)	40 (85.1)
Age, mean (SD)		55.6 (11.5)	52.5 (10.7)	55.1 (11.6)	52.8 (9.5)	54.4 (11.7)	55.9 (12.1)	56.3 (9.7)	56.0 (11.1)	51.6 (8.7)	56.2 (9.0)
With partner, n (%)		193 (83.6)	27 (81.8)	65 (79.3)	43 (81.1)	37 (75.5)	31 (81.6)	42 (82.4)	109 (81.3)	16 (69.6)	36 (76.6)
BMI, mean (SD)		26.0 (5.0)	27.2 (7.3)	26.2 (4.3)	25.7 (5.0)	25.3 (4.0)	26.1 (3.5)	26.1 (3.6)	25.4 (4.7)	24.8 (3.1)	25.4 (3.9)
**Education, n (%)**											
	Low	76 (32.9)	13 (39.4)	23 (28.1)	12 (22.6)	15 (30.6)	12 (31.6)	18 (35.3)	42 (31.3)	9 (39.1)	13 (27.7)
	Medium	76 (32.9)	12 (36.4)	31 (37.8)	20 (37.7)	20 (40.8)	13 (34.2)	18 (35.3)	44 (32.8)	7 (30.4)	14 (29.8)
	High	79 (34.2)	8 (24.2)	28 (34.2)	21 (39.6)	14 (28.6)	13 (34.2)	15 (29.4)	48 (35.8)	7 (30.4)	20 (42.6)
Working at baseline, n (%)		122 (52.8)	20 (60.6)	40 (48.8)	38 (71.7)	28 (57.1)	18 (47.4)	27 (52.9)	70 (52.2)	13 (56.5)	26 (55.3)
**Type of cancer, n (%)**											
	Breast	162 (70.1)	24 (72.7)	55 (67.1)	40 (75.5)	36 (73.5)	27 (71.1)	41 (80.4)	94 (70.2)	18 (78.3)	32 (68.1)
	Colon	29 (12.6)	4 (12.1)	10 (12.2)	4 (7.6)	6 (12.2)	5 (13.2)	2 (3.9)	19 (14.2)	3 (13.0)	9 (19.2)
	Other	40 (17.3)	5 (15.2)	17 (20.7)	9 (16.9)	7 (14.3)	6 (15.8)	8 (15.7)	21 (15.7)	2 (8.7)	6 (12.8)
Had cancer before, n (%)		24 (10.4)	5 (15.2)	8 (9.8)	3 (5.7)	4 (8.2)	3 (7.9)	5 (9.8)	13 (9.7)	2 (8.7)	5 (10.6)
**Treatment, n (%)**											
	Surgery, chemo, radio	86 (37.2)	11 (33.3)	37 (45.1)	20 (37.7)	20 (40.8)	18 (47.4)	22 (43.1)	53 (39.6)	11 (47.8)	22 (46.8)
	Surgery, chemo	61 (26.4)	11 (33.3)	17 (20.7)	16 (30.2)	16 (32.7)	9 (23.7)	12 (23.5)	35 (26.1)	7 (30.4)	15 (31.9)
	Surgery, radio	46 (19.9)	5 (15.2)	15 (18.3)	11 (20.8)	10 (20.4)	5 (13.2)	11 (21.6)	26 (19.4)	3 (13.0)	8 (17.1)
	Other	38 (16.5)	6 (18.2)	13 (15.9)	6 (11.3)	3 (6.1)	6 (15.8)	6 (11.8)	20 (14.9)	2 (8.7)	2 (4.3)
	Weeks since completion treatment, mean (SD)	25.1 (13.5)	27.1 (15.6)	24.1 (14.4)	22.3 (13.7)	25.3 (13.6)	26.5 (12.9)	23.7 (13.6)	25.0 (13.1)	22.1 (13.2)	25.4 (3.9)
Having comorbidities, n (%)		62 (26.8)	10 (30.3)	25 (30.5)	14 (26.4)	12 (24.5)	10 (26.3)	15 (29.4)	34 (25.4)	7 (30.4)	8 (17.0)
Using aftercare, n (%)		145 (62.8)	25 (75.8)	46 (56.1)	38 (71.7)	32 (65.3)	29 (76.3)	31 (60.8)	83 (61.9)	12 (52.2)	29 (61.7)

**Table 3:** Provided MRA and subsequent module use.

Module	Red			Orange			Green		
		Followed module, %			Followed module, %			Followed module, %	
	%	yes	no	%	yes	no	%	yes	no
Fatigue	34.6	58.8	41.3	19.1	38.6	61.4	46.3	16.8	83.2
Return to work	3.9	55.6	44.4	18.2	52.4	47.6	77.9	14.4	85.6
Mood	1.7	25	75	28.6	30.3	69.7	69.7	17.4	82.6
Relationships	11.7	25.9	74.1	19.1	27.3	72.7	69.3	11.8	88.1
Physical activity	5.2	25	75	35.9	37.4	62.7	58.9	12.5	87.5
Diet	53.3	50.4	49.6	42.9	68.7	31.3	3.9	44.4	55.6
Smoking	11.7	48.2	51.9	3.1	42.9	57.1	85.3	3.6	96.5

**Table 4:** Relationship between the MRA and module use (chi-square tests; df=1).

Module (yes/no)	Red compared to orange			Red compared to green			Orange compared to green		
	*X* ^2^	*P*	Odds ratio (95% CI)	*X* ^2^	*P*	Odds ratio (95% CI)	*X* ^2^	*P*	Odds ratio (95% CI)
Fatigue	4.599	.032^a^	2.262 (.99-5.16)	35.485	.000^a^	7.042 (3.12-14.69)	8.332	.004^a^	3.113 (1.30-7.37)
Return to work	0.030	.863	1.136 (.21-6.56)	10.565	.001^a^	7.404 (1.46-39.25)	28.920	.000^a^	6.515 (2.92-14.47)
Mood	0.050	.822	.767 (.01-10.27)	0.156	.693	1.583 (.03-20.50)	4.680	.031^a^	2.065 (1.00-4.21)
Relationships	0.016	.901	.933 (.26-3.11)	3.810	.051	2.597 (.81-7.49)	6.349	.012^a^	2.783 (1.11-6.73)
Physical activity	0.696	.404	.186 (.00-1.48)	1.474	.225	2.333 (.37-10.57)	18.60	.000^a^	4.173 (2.02-8.74)
Diet	7.553	.006^a^	.463 (.26-.83)	0.119	.730	1.27 (.26-6.71)	2.182	.140	2.742 (.54-14.67)
Smoking	0.063	.803	1.238 (.17-10.06)	58.075	.000^a^	25.204 (7.67-85.09)	22.400	.000^a^	20.357 (2.40-141.94)

^a^Statistically significant result.

### Appreciation

From the 231 participants who had access to the KNW intervention, 182 responded to the questions concerning appreciation after 6 months. The overall appreciation of the KNW was high (mean 7.5, SD 1.2) (Table 5). In general, the overall KNW was rated more positively among module users compared to non-module users. Ratings of the separate modules ranged from 6.4 (satisfactory) for the residual symptoms module to 8 (good) for smoking module. Personal relevance ranged from 2.9 to 3.5 (a little bit relevant to relevant). The ratings for comprehensibility, usefulness, and recommendation to other cancer survivors were all positive and very uniform (Table 5).

**Table 5:** Appreciation of KNW after 6 months.

		Overall	No module	Fatigue	Return to work	Mood	Relationships	PA	Diet	Smoking	Residual symptoms
Overall KNW (1-10), mean (SD)		7.5 (1.2)	7.1 (2.0)	7.6 (1.1)	7.6 (1.1)	7.4 (1.0)	7.4 (1.0)	7.6 (1.1)	7.5 (1.0)	7.8 (1.2)	7.4 (1.1)
Modules (1-10)^a^, mean (SD)				7.3 (1.3)	7.0 (1.3)	7.5 (1.2)	7.2 (0.8)	7.7 (1.1)	7.6 (1.0)	8 (1.3)	6.4 (1.9)
**Subquestions on content (1-5) ^b^, mean (SD)**											
	Understandable?	4.3 (0.6)	4.1 (1.0)	4.4 (0.5)	4.4 (0.5)	4.3 (0.5)	4.5 (0.5)	4.4 (0.5)	4.4 (0.5)	4.3 (0.5)	4.4 (0.5)
	Useful?	3.7 (0.8)	3.7 (1.1)	3.8 (0.8)	3.7 (0.8)	3.7 (0.8)	3.7 (0.8)	3.7 (0.7)	3.7 (0.8)	3.8 (0.9)	3.4 (0.9)
	Personal relevant?	3.2 (0.9)	2.9 (1.2)	3.4 (0.8)	3.3 (0.7)	3.2 (0.9)	3.4 (0.9)	3.5 (0.7)	3.2 (0.8)	3.3 (0.9)	3.3 (0.9)
	Recommendable to fellow survivors?	3.9 (1.0)	3.6 (1.1)	3.9 (1.0)	3.9 (1.0)	3.8 (1.0)	3.7 (1.0)	4 (1.0)	3.9 (1.0)	4.1 (0.9)	3.8 (1.0)

^a^No module n=18, fatigue n=47, return to work n=27, mood n=13, relationships n=11, PA n=28, diet n=77, smoking n=6, residual symptoms n=14.

^b^No module n=18, fatigue n=67, return to work n=46, mood n=45, relationships n=34, PA n=45, diet n=115, smoking n=18, residual symptoms n=39.

### Predictors of a Higher Number of Modules Used

Using a higher number of modules was predicted by a higher number of red/orange MRA (*β=*.136, *P=*.009), and by a higher perceived personal relevance (*β=*.150, *P=*.014). Moreover, having a partner was significantly related with a lower number of modules used (*β*=-.256, *P*=.044) (Multimedia Appendix 3).

### Predictors of a Higher Appreciation of KNW Overall

A higher appreciation with the overall KNW was significantly predicted by a higher perceived personal relevance (*β*=.623, *P*=.000) (Multimedia Appendix 4). None of the demographic and cancer-related variables, or the number of red/orange MRA, or number of modules used predicted a higher overall appreciation of the KNW intervention.

### Predictors of a Higher Perceived Personal Relevance

None of the demographic and cancer-related characteristics significantly predicted the perceived personal relevance of the KNW content, indicating that the KNW content was rated comparably personal relevant among individuals with different demographic and cancer-related characteristics (Multimedia Appendix 5).

## Discussion

### Principal Findings

This process evaluation of the Web-based KNW evaluated the automated guidance toward the KNW modules and subsequent module use, and the appreciation of this intervention. Despite the noncommittal nature of the KNW, more than 85% of the participants used one or more of the eight modules, and there was clear interest in all eight modules. This result confirms the need for wide-ranging support among early cancer survivors. Interestingly, automated referrals to specific modules were related to a higher number of modules used. Moreover, the complex KNW was highly appreciated and perceived as personal relevant by early cancer survivors.

The MRA aimed to guide the respondents toward the appropriate modules by giving feedback about current problem areas and needs. Cancer survivors might not have noticed some of these needs, and the MRA may have raised awareness about these topics. The importance of increasing awareness is theoretically grounded as described by Weinstein and Sandman [55] in their Precaution Adoption Process Model. That model includes a sequence of five stages within behavior change: “unaware of the issue,” “aware of the issue but not personally engaged,” “engaged and deciding what to do,” “planning to act but not yet having acted,” and “acting.” Prior research confirmed that a considerable number of colorectal cancer survivors were unaware of healthy diet recommendations, and older cancer survivors reported being less aware of the beneficial effects of a healthy lifestyle [56,57]. In addition, research revealed that cancer survivors might be less aware of available psychosocial support and solutions to psychosocial problems, while, for example, addressing maladaptive illness perceptions and adopting a more adaptive self-management may lead to better health outcomes [58,59]. Consequently, curiosity about available self-management support needs to be encouraged [8]. In accordance with the I-Change Model, the MRA could increase knowledge about the current level of well-being, psychosocial conditions, and lifestyle behavior. Besides that, the MRA could elevate the risk perception and may serve as a cue to action with regard to the relevant topics, given that the solutions to the problems are provided (relevant self-management module) [50]. These awareness/solution triggers might positively influence the motivation and intention to perform desired behavior, which is in line with the findings of Walthouwer et al [60], who identified awareness as an important moderator in the relationship between psychosocial determinants and specific dietary behavior (eating in moderation) in the general population. Results in our study illustrate that these awareness/solution triggers are most likely to be followed when a red or orange MRA was provided. Thus, the MRA successfully referred those respondents with elevated as well as severe complaints and/or needs. However, this did not apply for fatigue because highly fatigued respondents (red MRA) were more likely to use the fatigue module compared to participants with less fatigue (orange MRA). Additionally, with regard to diet, results might indicate that especially those who were already engaged more in a healthy diet were more likely to use the diet module. Furthermore, the topic diet could be of general interest to the participants, while the topic fatigue might be most interesting for participants with specific complaints. Consequently, the MRA may be a meaningful intervention component to increase motivation, subsequent module use, and problem-solution, while MRA adherence might be related to the specific behavior. Using topic-specific KNW modules has shown to be effective in decreasing fatigue, depressive feelings, and was beneficial in increasing moderate physical activity and fruit and fish consumption [16,17].

Within the KNW, participants were referred on average to 2.9 modules, while on average 2.1 modules were used. The appreciation rates were high, and the results showed that a higher number of modules used did not contribute to a higher appreciation. However, a higher perceived personal relevance did contribute to a higher appreciation. This is in line with Wilson et al [61] reporting that a moderate number of recommendations in multiple behavior interventions might produce the highest level of change, while engagement with a higher number of recommendations might be too demanding. Within the KNW, respondents were allowed to make their own choices, despite the provided MRA. Prior research confirms that the possibility to choose within multiple behavior interventions may prevent high attrition rates and could improve intervention outcomes [31,32,62]. Offering wide ranging support in combination with personalized referral to relevant topics and the possibility to choose might prevent overload. Donkin et al [33] support this suggestion by reporting that a certain level of usage might be needed to obtain benefit from an online intervention for depression. However, after reaching a point of therapy saturation, little or no additional program gains might be expected. This is in line with a Web-based study among cancer survivors and with another Web-based obesity prevention study among the general population, which reported that more intervention use did not result in better intervention outcomes [28,63]. Using a higher number of modules may not be necessary for all users to benefit most from the KNW. Our results revealed that having no partner was related to the use of a higher number of modules, and participants who were in greater need of support (higher number of red/orange MRA) indeed used a higher number of modules. This is consistent with the findings of Borosund et al [28], who reported that, in particular, cancer survivors with low levels of social support and a high illness burden used self-management components of a Web-based illness management support system. Furthermore, higher perceived personal relevance was related to using a higher number of modules, which might be explained by receiving a higher amount of computer-tailored content within the modules. The overall KNW was highly appreciated with an average grade of 7.5, indicating an appreciation from very satisfactory to good. The low variability (SD 1.2) indicates a considerably unanimous positive rating 6 months after getting access to the KNW. Results from our study indicate that perceived personal relevance might be a key component to explain a higher appreciation. Computer tailoring was applied within the KNW in order to create personal relevant feedback. Since perceived personal relevance could not be predicted by demographic and cancer-related characteristics, we can conclude that the tailoring of information worked well. In comparison, the overall satisfaction of a generic fully automated Web-based self-management intervention for breast cancer survivors was mean 7 (SD 1.2) [24]. In addition, the overall appreciation of a Web-based weight management intervention for overweight adults was mean 6.6, and the overall appreciation of a Web-based text- and video-tailored intervention for smoking cessation in the general population was mean 6.45 (SD 1.62; scales ranged from 1-10) [53,54]. The overall appreciation ratings of KNW module users were more positive than the ratings of module non-users, although the module non-users were still quite positive in their ratings. In addition to the modules, the KNW has a user forum and participants received monthly emails inviting them to visit generic monthly news items. Filling out the screening questionnaire and follow-up questionnaires, combined with receiving personalized feedback on problem areas (by the MRA), as well as the additional KNW features, might already have raised awareness and provided other valuable information to achieve benefits among module non-users. Overall, the high appreciation rate indicates that the broad design and tailored information of the KNW seem to fit well with the needs of early cancer survivors (in which, breast cancer survivors were overrepresented).

### Limitations

Some limitations need to be addressed. First, providing data on completion of the separate themes and specific activities within the modules, and on completion of the evaluation sessions was not possible due to the module design. This information might be interesting for future studies; therefore, we recommend future interventions to study in more detail participation of intervention modules. Second, within our study, it was not possible to compare the relationships between the MRA and module use to a control group not receiving the MRA. Consequently, these associations need to be interpreted with caution, as it is conceivable that without the MRA, some of the same modules would have been used. Future experimental research might explore the specific effects of a similar automated referral system on subsequent choices. Third, this eHealth intervention requires respondents to have computer skills and health literacy, such as competence at accessing, understanding, appraising, and applying the health information provided [64]. However, since eHealth literacy was not assessed in this study, it is not possible to estimate the extent to which this might have influenced initial recruitment and the use and appreciation of the KNW. Fourth, mainly middle-aged, female breast cancer survivors who scored fairly well on QoL and depression participated, which might be too selective a group to represent the general cancer survivor population. During recruitment, mainly breast cancer outpatient clinics participated. Five-year survival rates of breast cancer are relatively high [6]. Unless mostly females with higher socioeconomic status are reached in Web-based interventions in general, interpretations of these findings should be viewed with caution [8].

### Conclusion

The general KNW and the KNW modules were substantially used and highly appreciated by early cancer survivors, thus confirming the need for wide-ranging support among this target group. Results indicate that the MRA may be seen as a meaningful key component of the fully automated KNW intervention by guiding users to follow a preferred selection of modules, given their current complaints and identified needs. Moreover, the overall intervention and separate modules were highly appreciated, which could be explained by a higher perceived personal relevance. We can conclude that computer tailoring worked well and that the range of topics, design, and personalized information suited the needs of early cancer survivors. This process evaluation adds meaningful information on the use and appreciation of Web-based cancer aftercare interventions and confirms that the KNW offers valuable and appropriate support for early cancer survivors to complement usual cancer aftercare and may serve as a first step in a stepped-care approach.

## Appendix

Multimedia Appendix 1 Determination of the Module Referral Advice categories (red, orange, green).

Multimedia Appendix 2 Overview of cancer diagnoses among the KNW sample.

Multimedia Appendix 3 Predictors of a higher number of followed KNW modules (N=182).

Multimedia Appendix 4 Predictors of a higher appreciation of KNW (N=182).

Multimedia Appendix 5 Predictors of a higher perceived personal relevance of KNW content (N=182).

## Abbreviations

BMI: Body Mass Index
CaSUN: Cancer Survivors’ Unmet Needs questionnaire
CIS: Checklist Individual Strength
HADS: Hospital Anxiety and Depression Scale
KNW: Kanker Nazorg Wijzer (Cancer Aftercare Guide)
MAC: Mental Adjustment to Cancer Scale
MRA: Module Referral Advice
PA: physical activity
QoL: quality of life
RCT: randomized controlled trial
SQUASH: Short Questionnaire to Assess Health Enhancing Physical Activity
SSL-D: Social Support List‒Discrepancy Subscale
