# Abstract and Metadata Correction of: Use and Appreciation of a Tailored Self-Management eHealth Intervention for Early Cancer Survivors: Process Evaluation of a Randomized Controlled Trial

**DOI:** 10.2196/jmir.6564

**Published:** 2016-09-13

**Authors:** Iris M Kanera, Roy A Willems, Catherine AW Bolman, Ilse Mesters, Victor Zambon, Brigitte CM Gijsen, Lilian Lechner

**Affiliations:** ^1^ Faculty of Psychology and Educational Sciences Open University of the Netherlands Heerlen Netherlands; ^2^ Research Institute CAPHRI Department of Epidemiology, Optimizing Patient Care Maastricht University Maastricht Netherlands; ^3^ Department of Urology Zuyderland Medical Center Heerlen and Sittard-Geleen Netherlands; ^4^ Netherlands Comprehensive Cancer Organisation (IKNL) Utrecht Netherlands

The authors of “Use and Appreciation of a Tailored Self-Management eHealth Intervention for Early Cancer Survivors: Process Evaluation of a Randomized Controlled Trial” (J Med Internet Res 2016; 18(8):e229) have overlooked errors in the abstract during the proofreading process. The method section of the abstract was missing, the results section was displayed under ‘methods’, and the conclusion was displayed twice, under ‘results’ as well as under ‘conclusions’. The methods section in the abstract need to be the following:

Methods: This process evaluation was conducted as part of a randomized controlled trial. Early cancer survivors with various types of cancer were recruited from 21 Dutch hospitals. Data from online self-report questionnaires and logging data were analyzed from participants allocated to the intervention condition. Chi-square tests were applied to assess the adherence to the module referral advice, negative binominal regression analysis was used to identify predictors of module use, multiple linear regression analysis was applied to identify predictors of the appreciation, and ordered logistic regression analysis was conducted to explore possible predictors of perceived personal relevance.

This section needs to be added under ‘methods’, the results needs to be displayed under ‘results’, and the conclusion needs to displayed under ‘conclusions’.

Furthermore, the degrees of the authors were corrected. As per AMA Style Guide, only the highest degrees should be presented, thus we deleted "BSc" from the authors Kanera and Gijsen. In addition, the degree of author Brigitte CM Gijsen (Master of Science in Public Health) was corrected from "MPH" to "MSc" in order to provide uniformity in degrees.

The affiliations of the authors have not been changed. This correction has been made in the online version of the paper on the JMIR website on September 13, 2016, together with publishing this corrigendum. 

A correction notice has been sent to PubMed, and the publication was resubmitted to Pubmed Central and other full-text repositories.

