# Supplementary material for: Use and Appreciation of a Tailored Self-Management eHealth Intervention for Early Cancer Survivors: Process Evaluation of a Randomized Controlled Trial
Source: J Med Internet Res. 2016 Aug 23;18(8):e229. doi: 10.2196/jmir.5975 (PMC5013245; doi:10.2196/jmir.5975)
Supplement: Multimedia Appendix 1 [file jmir_v18i8e229_app1.pdf]

Determination of the Module Referral Advice-categories (red, orange, green)

The Module Referral Advice (MRA) concerning return to work was based on four items from the extended Cancer Survivors' Unmet Needs questionnaire (CaSUN) [4,37,38], that were formulated as follows: 1) "I need help to make adjustments to my job or to find a new job", 2) "I need help to find out details about receiving financial support", 3) "I need help with carrying out my work", 4) "I need information about rules and legislation on returning to work". Respondents indicated whether they have no need, a met need, or an unmet need. Strength of unmet needs is rated as weak, moderate, or strong. The item range was 0-5, with a total score range of 0-20. Scores ranging from 0-3 (no actual need) were classified into a green MRA, scores ranging from 3-12 (some needs) into an orange MRA, and scores higher than 12 (strong needs) were classified into a red MRA.

The 'fatigue' MRA was based on eight items of the subscale subjective fatigue of the Checklist Individual Strength (CIS) (e.g., "I am feeling tired") with an item range of 1-7 (score range of 8-56) [36]. The classification into a green, orange, and red MRA corresponded to the cut-off scores of the CIS subscale subjective fatigue: < 27 = normal (green), 27-35 = elevated (orange), > 35 = severe.

For the MRA classification of social relationship issues, the discrepancy subscale from the 6-item version of the Social Support List (SSL-D) was used [41]. The items ranged from 1-4 with a total score range of 6-24 and measures the extent to which the respondent is experiencing a lack of social support. Green, orange, and red categories were constructed by combining the SSL-D with two items from the extended CaSUN. Scores of SSL-D ≤ 7 ("social support is just right") resulted in a green MRA, scores of 8 and 9 on the SSL-D (experiencing two or three problems with social support) in combination with one of the needs concerning fertility or sexuality resulted in an orange MRA, and scores of 10 or higher

on the SSL-D (experiencing at least four problems with social support) resulted in a red MRA.

The MRA with regard to mood was based on responses to the Hospital Anxiety and Depression Scale (HADS) [39] and the dimension 'negative adjustment' of the Mental Adjustment to Cancer Scale (MAC) [40]. Concerning the HADS, seven items for anxiety and seven items for depression were measured on a 4-point scale with scores ranging from 0-21. Negative mental adjustment comprises 16 items of the MAC as described by Watson et al. [40] with scores ranging from 16-64, and a cut-off score of 36 indicating problematic functioning. Scores lower than 8 on the subscale anxiety and the subscale depression on the HADS, and a MAC score of 36 or lower resulted in a green MRA, indicating no/low psychological distress and no/low problematic functioning. A MAC score higher than 36 in combination with a score of 8-15 on one of the HADS subscales was categorized into an orange MRA. When the scores on one of the HADS subscales were higher than 15, or when both subscale scores were higher than 15, a red MRA was provided.

The MRA for physical activity (PA) and diet was constructed according to the lifestyle recommendations of the World Cancer Research Fund/American Institute for Cancer Research (WCRF/AICR) and the American Cancer Society [46,47]. The classification criteria are displayed in Table 1. PA was assessed by applying the validated 11-item self-report Short Questionnaire to Assess Health Enhancing Physical Activity (SQUASH) [42,43]. The number of days a week of PA (≥30 minutes of moderate intensity), the average number of minutes per day, and the intensity (light, moderate, vigorous) were rated for activities during commuting, work, household, leisure time, and sports [17].

Dietary behavior was measured by using 14 items from the Dutch Standard Questionnaire on Food Consumption to assess vegetable, fruit, fish, whole-grain bread,

oatmeal, cereal, potato, whole-grain rice, and whole-grain pasta consumption [44,17]. A green MRA was provided when respondents met four out of five dietary recommendations as displayed in Table 1. When reporting that two or three out of five conditions were met an orange MRA was provided, and respondents who met one or none of the five conditions received a red MRA.

The ‘smoking’ MRA was based on current and former smoking behavior, which was measured by using three standardized questions from Dutch Measuring Instruments for Research on Smoking and Smoking Cessation [45,17]. Respondents who had never smoked before and former smokers who quit smoking prior to the cancer diagnosis received a green MRA, former smokers who quit when the cancer was diagnosed received an orange MRA, and current smokers received a red MRA.
