# Supplementary material for: Use and Appreciation of a Tailored Self-Management eHealth Intervention for Early Cancer Survivors: Process Evaluation of a Randomized Controlled Trial
Source: J Med Internet Res. 2016 Aug 23;18(8):e229. doi: 10.2196/jmir.5975 (PMC5013245; doi:10.2196/jmir.5975)
Supplement: Multimedia Appendix 2 [file jmir_v18i8e229_app2.pdf]

Table 3. Overview of cancer diagnoses among the KNW sample, N = 231

Type of cancer	N	%
Breast	162	70.13
Colon	29	12.55
Non-Hodgkin's lymphoma	10	4.33
Lung	5	2.16
Uterus	4	1.73
Prostate	3	1.30
Esophagus	3	1.30
Kidney	3	1.30
Ovary	2	0.87
Vulva	2	0.87
Testicle	2	0.87
Cervix	1	0.43
Thyroid	1	0.43
Stomach	1	0.43
Bladder	1	0.43
Hodgkin's lymphoma	1	0.43
Multiple Myeloma	1	0.43
