# Supplementary material for: Use and Appreciation of a Tailored Self-Management eHealth Intervention for Early Cancer Survivors: Process Evaluation of a Randomized Controlled Trial
Source: J Med Internet Res. 2016 Aug 23;18(8):e229. doi: 10.2196/jmir.5975 (PMC5013245; doi:10.2196/jmir.5975)
Supplement: Multimedia Appendix 3 [file jmir_v18i8e229_app3.pdf]

Table 6. Predictors of a higher number of followed modules of the *Kanker Nazorg Wijzer*, (Cancer Aftercare Guide, KNW), N=182

	Higher number of followed modules (0-8)		
Variable	Beta	SE [95% CI]	*P*
Female gender	.068	.235 [-.39; .53]	.771
Age	.030	.066 [-.10; .16]	.650
Marital status: with partner	-.256	.127 [-.50; -.01]	**.044**
Being employed: *yes*	.177	.102 [-.08; .41]	.192
Education level (low=ref)			
Medium	.135	.123 [-.11; .38]	.274
High	.165	.127 [-.08; .41]	.192
Breast cancer (other=ref)	.035	.212 [-.38; .45]	.867
Primary cancer treatment (other= ref)			
Surgery & radiation	.147	.181 [-.31; .40]	.797
Surgery & chemo	.273	.183 [-.09; .63]	.163
Surgery & chemo & radiation	.251	.168 [-.08; .58]	.135
Number of weeks after completing treatment	.016	.048 [-.08; .11]	.730
Participating in aftercare: *yes*	-.076	.108 [-.29; .14]	.481
Having co-morbidities: *yes*	-.054	.122 [-.29; .19]	.657
BMI	-.058	.057 [-.17; .05]	.311
Number of orange/red MRA (0-7)	.136	.053 [.03; .24]	**.009**
Perceived personal relevance (1-5)	.150	.061 [.03; .27]	**.014**
Constant	.664	.316 [.04; 1.28]	.036
Pseudo R^2	.051		
Wald Chi-square (15)	51.48		.000

Note: Negative binomial regression was used. Beta = regression coefficient.
Abbreviations: ref: reference group; BMI: Body Mass Index; MRA: module referral advice
