# Supplementary material for: Use and Appreciation of a Tailored Self-Management eHealth Intervention for Early Cancer Survivors: Process Evaluation of a Randomized Controlled Trial
Source: J Med Internet Res. 2016 Aug 23;18(8):e229. doi: 10.2196/jmir.5975 (PMC5013245; doi:10.2196/jmir.5975)
Supplement: Multimedia Appendix 4 [file jmir_v18i8e229_app4.pdf]

Table 7. Predictors of a higher appreciation of the of the *Kanker Nazorg Wijzer*, (Cancer Aftercare Guide, KNW), N=182

	Appreciation (1-10)		
Variable	Beta	SE [95% CI]	*P*
Female gender	-.223	.355 [-.92; .48]	.530
Age	.069	.117 [-.16; .30]	.557
Marital status: with partner	.359	.265 [-.17; .88]	.178
Being employed: *yes*	.123	.178 [-.25; .49]	.514
Education level (low=ref)			
Medium	-.074	.198 [-.47; .32]	.708
High	.160	.181 [-.20; .52]	.379
Breast cancer (other=ref)	.242	.347 [-.44; .93]	.488
Primary cancer treatment (other= ref)			
Surgery & radiation	-.005	.344 [-.68; .67]	.987
Surgery & chemo	.011	.272 [-.55; .53]	.967
Surgery & chemo & radiation	-.409	.284 [-.97; .15]	.152
Number of weeks after completing treatment	.016	.077 [-.14; .17]	.833
Participating in aftercare: *yes*	-.166	.196 [-.55; .22]	.397
Having co-morbidities: *yes*	.359	.182 [-.00; .72]	.051
BMI	-.013	.018 [-.05; .02]	.474
Number of orange/red MRA (0-7)	-.158	.102 [-.36; .04]	.122
Number of followed modules (0-8)	.027	.080 [-.13; .18]	.738
Perceived personal relevance (1-5)	.623	.097 [.43; .82]	**.000**
R^2	.361		
F	1.013		.000

Note: Multiple linear regression was used. Beta = regression coefficient.
Abbreviations: ref: reference group; BMI: Body Mass Index; MRA: Module Referral Advice
