# Supplementary material for: Use and Appreciation of a Tailored Self-Management eHealth Intervention for Early Cancer Survivors: Process Evaluation of a Randomized Controlled Trial
Source: J Med Internet Res. 2016 Aug 23;18(8):e229. doi: 10.2196/jmir.5975 (PMC5013245; doi:10.2196/jmir.5975)
Supplement: Multimedia Appendix 5 [file jmir_v18i8e229_app5.pdf]

Table 8. Predictors of a higher perceived personal relevance of the KNW content, N=182

	Perceived personal relevance (1-5)		
Variable	Beta	SE [95% CI]	*P*
Female gender	-.027	.614 [-1.23; 1.18]	.964
Age	-.123	.221 [-.56; .31]	.578
Marital status: with partner	.246	.340 [-.54; 1.03]	.538
Being employed: *yes*	-.248	.342 [-.92; .42]	.468
Education level (low=ref)			
Medium	-.597	.369 [-1.32; .13]	.106
High	-.698	.368 [-1.42; .02]	.058
Breast cancer (other=ref)	-.403	.581 [-1.54; .74]	.488
Primary cancer treatment (other= ref)			
Surgery & radiation	-.303	.542 [-1.37; .76]	.576
Surgery & chemo	.600	.464 [-.31; 1.51]	.196
Surgery & chemo & radiation	.027	.479 [-.91; .97]	.955
Number of weeks after completing treatment	-.108	.140 [-.38; .17]	.439
Participating in aftercare: *yes*	.188	.332 [-.46; .84]	.570
Having co-morbidities: *yes*	-.054	.328 [-.70; .59]	.868
BMI	.013	.149 [-.28; .30]	.933
Pseudo R^2	.031		
Chi^2	14.58		.407

Note: Ordered logistic regression was used. Beta = regression coefficient.
Abbreviations: ref: reference group; BMI: Body Mass Index; MRA: Module Referral Advice
